# Dyadic Discrimination and Purpose in Life Among Midlife and Older Black Couples

**DOI:** 10.1093/geroni/igaf122.433

**Published:** 2025-12-31

**Authors:** Heather Farmer, Jeffrey Stokes

**Affiliations:** University of Delaware, Newark, Delaware, United States; University of Massachusetts Boston, Boston, Massachusetts, United States

## Abstract

Marriage is among the most central and closest relationships in adulthood. Psychosocial risks and resources are often shared, though dyadic processes in Black couples are underexplored. Given the interdependent nature of marriage, discrimination may jeopardize resilience resources, such as purpose in life, for the individual experiencing it and vicarious discrimination could affect their partner’s purpose as well. Moreover, diminished purpose in life may be “contagious” among older Black spouses, particularly those who lack structural protective factors such as educational attainment. We explore the dyadic associations between discrimination and purpose in life among 180 opposite-gender Black couples ages 50+ in the 2016-2020 waves of the Health and Retirement Study. We estimate the individual and dyadic associations between discrimination and purpose using longitudinal structural equation models, controlling for age, education, employment, and marital quality. We then included an interaction term [partner’s education x purpose] to the model. Results showed that individual-level discrimination was significantly associated with lower purpose only in women (b = -0.21, p<.01), and that vicarious discrimination was not associated with purpose. We found that a spouse’s purpose undermined one’s own purpose only when the spouse had low educational attainment. Findings provide preliminary evidence of interpersonal mechanisms that may hinder psychological well-being among Black couples. Shared psychosocial experiences in marriage may be tied to health in Black populations, for better or worse, and deserve more scholarly attention. Therefore, considering purpose as a shared, rather than individual, resource may lead to effective interventions aimed at supporting purpose to enhance well-being.

